# Enhanced Catalytic Performance of Ag NP/0.95AgNbO_3_-0.05LiTaO_3_ Heterojunction from the Combination of Surface Plasma Resonance Effect and Piezoelectric Effect Using Facile Mechanical Milling

**DOI:** 10.3390/nano13222972

**Published:** 2023-11-18

**Authors:** Tianxiang Ren, Tufeng He, Zhenzhu Cao, Pengyue Xing, Xinglong Teng, Guorong Li

**Affiliations:** 1 Chemical Engineering College, Inner Mongolia University of Technology, Hohhot 010051, China; 20211800171@imut.edu.cn (T.R.); 20211800184@imut.edu.cn (T.H.); 20221100176@imut.edu.cn (P.X.); 20221800182@imut.edu.cn (X.T.); 2 Engineering Research Center of Large Energy Storage Technology, Ministry of Education, Inner Mongolia University of Technology, Hohhot 010051, China; 3 Key Laboratory of Inorganic Function Material and Device, Shanghai Institute of Ceramics, Chinese Academy of Sciences, Shanghai 200050, China; grli@mail.sic.ac.cn

**Keywords:** SPR effect, piezoelectric effect, photocatalysis, visible light, decomposition

## Abstract

An internal built electric field can suppress the recombination of electron–hole pairs and distinctly enhance the catalytic activity of a photocatalyst. Novel t-Ag/0.95AgNbO_3_-0.05LiTaO_3_ heterojunction was prepared by reducing silver nanoparticles (Ag NPs) on the surface of the piezoelectric powder 0.95AgNbO_3_-0.05LiTaO_3_ (0.05-ANLT) using a simple mechanical milling method. The effects of milling time and excitation source used for the degradation of organic dye by heterojunction catalysts were investigated. The results demonstrate that the optimized 1.5-Ag/0.05-ANLT heterojunction removes 97% RhB within 40 min, which is 7.8 times higher than that of single piezoelectric catalysis and 25.4 times higher than that of single photocatalysis. The significant enhancement of photocatalytic activity can be attributed to the synergistic coupling of the surface plasmon resonance (SPR) effect and the piezoelectric effect.

## 1. Introduction

The continuous utilization of fossil fuels results in serious environmental pollution and energy shortage, which blocks the sustainable development of contemporary society. Among these problems, water pollution has been further exacerbated by the extensive release of chemical dyes from the textile and printing sectors [1,2,3]. Photocatalysis excited by sunlight can decompose organic pollutants in water and provide a low-cost and environmental friendly solution [4,5,6]. However, the limited light response of conventional single-component photocatalysts and the high electron–hole pair recombination rate greatly hinder their quantum efficiency, thereby imposing significant constraints on their practical applications [7]. Many methods have been proposed to overcome these disadvantages [8,9,10,11]. Due to its ability to broaden the light absorption spectrum and mitigate electron–hole recombination, heterojunction has drawn more and more attention in recent years [12].

Unfortunately, the heterojunction can only drive the photoinduced charges near the junction region to take part in the photocatalysis reaction, which means that the electron and hole in the bulk of the semiconductor have been left to recombine. The piezopotential induced by external stress in piezoelectric has been proven to separate excited charges in deep regions [13]. For instance, zinc oxide (ZnO) nanowires reduced the recombination of electron–hole pairs under external pressure and enhanced the degradation of methylene blue (MB) [14]. 

It is hypothesized that the combination of heterojunction and piezopotential could further enhance the activity of the catalyst. Nevertheless, the piezo-photocatalysis performance of common piezoelectric is hindered by the low visible light response from their large band gap (>3 eV) [15]. Currently, heterostructure construction [16], defect engineering [17], and chemical modification [18] have been used to enhance the catalytic efficacy of piezoelectric materials. It is well known that the design of morphotropic phase boundaries (MPBs) greatly improved the electrical properties of piezoelectric materials [19]. The coexistence of multiple phases provides more directions for polarization, which facilitates polarization rotation. The piezoelectric potential is directly determined by the piezoelectric constant according to the following equation: V = d_33_ (SYL)/ε_0_ε, where d_33_ is the piezoelectric constant, L is the original thickness, Y is Young’s modulus of the piezoelectric, ε_0_ is the permittivity of free space, and ε is the relative dielectric constant. Exceptional piezo-photocatalytic performance has been observed near the morphotropic phase boundary in BiPrFeMnO_3_ nanofibers [20], Sm-doped PMN-PT [21], and KNN [22] powder.

AgNbO_3_, with a band gap of approximately 2.8 eV, shows notable visible light absorption [23]. The distinctive d^10^ electronic structure and plasmonic resonance effect of AgNbO_3_ make it a versatile catalyst for hydrogen production [24,25] and the degradation of organic pollutants [26,27]. On the other hand, strong ferroelectric and piezoelectric properties can be achieved via the formation of a solid solution between AgNbO_3_ and other ferroelectrics (Ag_1−x_K_x_NbO_3_ [28], (Ag_1−x_Li_x_)NbO_3_ [29], and (1 − x)AgNbO_3_-xLiTaO_3_ [30]). In addition, the manipulation of its ferro/piezoelectric properties has been established as a significant approach to enhancing the photocatalytic efficacy of AgNbO_3_ [31,32].

The plasmon resonance effect (SPR) from nano noble metal (Au, Ag, Pt) particles on surfaces can produce a strong localized electromagnetic field and enhance the efficiency of electron and hole separation in semiconductors [33,34]. Larger K^+^ substitution for Ag^+^ transforms AgNbO_3_ from an antiferroelectric to a ferroelectric state, building a substantial internal electric field. Additionally, a minute quantity of metallic silver can be generated at elevated levels of K^+^ doping. Combining the SPR and piezoelectric potential, the piezo-photocatalytic degradation has also been sharply enhanced [35]. At present, the methods of loading noble metal on semiconductors mainly include piezoelectric electrochemical deposition [36], photochemical reduction [37], and impregnation [38]. Mechanochemical synthesis through ball milling can avoid the use of hazardous organic solvents and external heating, shorten reaction times, and simplify the synthesis process [39]. Silver nanoparticles (Ag NPs) were deposited onto TiO_2_ via mechanical ball milling. Compared with pure TiO_2_, the degradation rate of methyl orange (MO) dye under UV irradiation by Ag/TiO_2_ heterojunction has been sharply increased (2.1 times) [40].

Recently, superior catalytic activity (1 − x)AgNbO_3_-xLiTaO_3_ solid solution near the antiferroelectric–ferroelectric (AFE-FE) phase boundary has been reported by our group [41]. It was found that the color of the fresh solid solution (light yellow) has changed to dark yellow during dry milling, which indicates that some reaction has occurred. In this study, t-Ag/0.95AgNbO_3_-0.05LiTaO_3_ (hereafter referred to as t-Ag/0.05-ANLT) piezo-photocatalyst was synthesized through a facile mechanical grinding. The piezo-photocatalytic degradation of the organic dye of this heterojunction has been investigated. Within 40 min, the removal rate of RhB by 1.5-Ag/0.05-ANLT heterojunction under light and ultrasonic vibration was 97%, which was 7.8 times that of piezoelectric catalysis alone and 25.4 times that of photocatalysis alone.

## 2. Materials and Methods

### 2.1. Materials

Analytical-grade niobium pentoxide (Nb_2_O_5_, 99%), lithium carbonate (Li_2_CO_3_, 98%), silver oxide (Ag_2_O, 99.8%), and tantalum pentoxide (Ta_2_O_5_, 99.9%), along with ethanol and rhodamine B (RhB), were procured from Sinopharm Chemical Reagent Corp. (Shanghai, China). The chemicals were employed in their original form without additional purification. Furthermore, all aqueous solutions were prepared using deionized water.

### 2.2. Preparation of the Samples

In this study, 0.05-ANLT powder was synthesized using a traditional solid-state reaction technique. In this process, a thorough mixing of Ag_2_O, Nb_2_O_5_, Li_2_CO_3_, and Ta_2_O_5_ was achieved in anhydrous ethanol for 30 min using a mortar. The resulting powders were then dried and calcined at a temperature of 900 °C for 6 h while maintaining a constant oxygen flow rate of 5 mL/min.

After that, a grinding process was used to form Ag NPs on the surface of 0.05-ANLT. Specifically, 0.2 g of 0.05-ANLT solid solution powder was subjected to grinding with zirconia balls in a mini-mill (MSK-SFM-12M, Hefei Kejing, Hefei, China), with the sample-to-ball mass ratio of 1:2. The 0.05-ANLT powders ground for 0.5, 1, 1.5, and 2 h were denoted as 0.5-Ag/0.05-ANLT, 1-Ag/0.05-ANLT, 1.5-Ag/0.05-ANLT, and 2-Ag/0.05-ANLT, respectively. During the grinding process, it is imperative to shield the sample from light.

### 2.3. Characterization

Powder X-ray diffraction (PXRD) was utilized to examine the composition of the milled powder. The measurement was conducted using Cu Ka radiation (Rigaku Corporation, Tokyo, Japan) with a voltage of 45 kV and a current of 200 mA. Ultraviolet-visible spectroscopy (UV-vis) measurement was performed using a UV-vis spectrophotometer (Hitachi UV-3600, Tokyo, Japan) equipped with an integrating sphere attachment. To further investigate the morphology of the samples, transmission electron microscope (TEM) and high-resolution TEM (HRTEM) (Talos F200X, FEI, Thermo Fisher Scientific, Waltham, MA, USA) were employed. The photoluminescence (PL) spectra were acquired via fluorescence spectroscopy (RF-5301PC, Shimadzu, Tokyo, Japan) at a stimulation wavelength of 365 nm.

### 2.4. Piezo-Photocatalytic Characterization

The piezo-photocatalytic activity of the t-Ag/0.05-ANLT catalyst was assessed through the degradation of RhB. This evaluation involved the utilization of 300 W Xenon lamp (CEL-HXF300, CEAULIGHT, Beijing, China), which was equipped with a UV cutoff filter to produce visible light above 420 nm. An ultrasonic cleaner (40 kHz and 300 W, KQ-300DE, Kunshan, China) was employed as an ultrasonic source. In this study, 100 mg of t-Ag/0.05-ANLT powder was dispersed into 100 mL RhB dye solution of 5 mg/L. To attain adsorption–desorption equilibrium, the mixture was agitated in the absence of light for one hour. Throughout the degradation process, approximately 3 mL of the solution was extracted in 10 min intervals and subsequently filtered through a 0.45-μm Millipore filter. The concentration of RhB was determined using a UV-Vis spectrometer (UVT6, Beijing Purkinje General Instrument Co., Ltd., Beijing, China) by measuring its characteristic absorbance at 554 nm. The suspension subjected to ultrasonic vibration was maintained at room temperature to prevent any heat-induced catalytic effects.

### 2.5. Detection of Active Species in Catalysis

To unveil the active species engaged in the degradation of RhB dye, radical capture experiments were conducted. Three distinct scavengers for holes (*h^+^*), superoxide ions (•O_2_^−^), and hydroxyl radicals (•OH), namely triethanolamine (TEOA), benzoquinone (BQ), and isopropyl alcohol (IPA) were employed correspondingly.

### 2.6. Electrochemical Performance Test

To measure the photocurrent, electrochemical impedance (EIS) and Mott–Schottky curve, we employed a conventional three-compartment cell consisting of a working electrode, a Pt wire counter electrode, and a reference electrode saturated with calomel. The electrolyte solution was 0.1 M Na_2_SO_4_. Xenon lamp light source (Zolix HPS-300XA, Beijing, China) was used to irradiate the electrochemical cell. The current and voltage signals were obtained using the I-t program of the electrochemical workstation (Chi660E, Chenhua, Shanghai, China). EIS measurements were conducted with an AC amplitude of 5 mV in a frequency range of 10^−2^ to 10^5^ Hz. The Mott–Schottky curves of the working electrode were recorded at frequencies of 1000 Hz using the same electrochemical workstation (Chi660E, Chenhua, Shanghai, China).

## 3. Results

**Figure 1:**
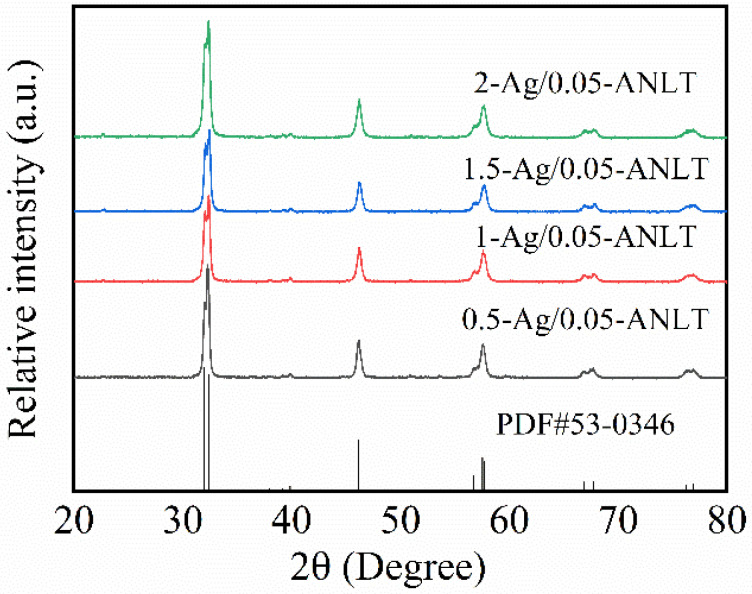
XRD patterns of t-Ag NP/0.05-ANLT samples after grinding for different times.

Figure 1 displays the X-ray diffraction (XRD) patterns of t-Ag NP/0.05-ANLT catalysts. Similar diffraction patterns have been observed for all powders after grinding for different times. These diffraction peaks match well with the standard card of 0.05-ANLT solid solution (JCPDS No. 53-0346) [30]. This finding confirms the high crystallinity of t-Ag/0.05-ANLT powders and indicates that the crystal structure of 0.05-ANLT solid solution remains unchanged basically after grinding. As reported in the literature, the powder of 0.05-ANLT is composed of orthorhombic (O) and rhombohedral (R) phases [35]. This composition has high piezoelectricity. However, the color of the powder is darkened, which indicates that some Ag might be reduced out [33]. The absence of diffraction peaks corresponding to Ag in the XRD pattern can be attributed to its extremely low concentration, which falls below the detection limit of X-ray. Further investigation is necessary to validate the presence of Ag on the surface of the 0.05-ANLT particle.

**Figure 2:**
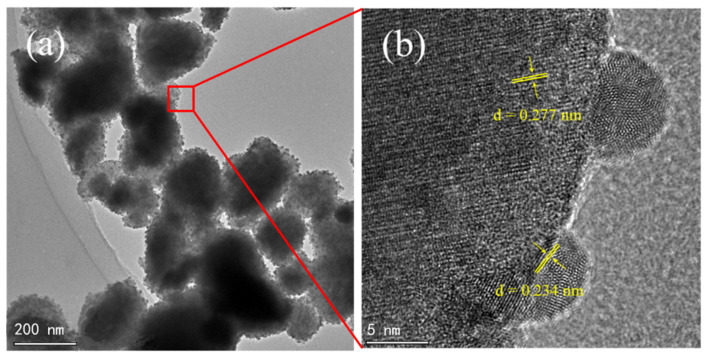
TEM and HRTEM images of 1.5-Ag/0.05-ANLT (**a**,**b**).

TEM and HRTEM results of 1.5-Ag/0.05-ANLT composite are shown in Figure 2. It is reported that a smooth and clean surface can be seen in fresh 0.05-ANLT. The size of the particles varies between 400 and 800 nm [41]. As seen from Figure 2a, the particle size decreases to 150–500 nm after mechanical grinding for 1.5 h. It should be stressed that some nanoparticles are uniformly dispersed on the surface of the large one. The lattice fringe widths of 0.234 nm and 0.277 nm (Figure 2b) correspond to the (111) plane of Ag (JCPDS No. 04-0783) and the (114) plane of AgNbO_3_ (JCPDS No. 52-0405), respectively. The size of Ag NPs is about 7 nm, and they are tightly anchored to the surface of 0.05-ANLT solid solution particle and form the heterojunction [42].

**Figure 3:**
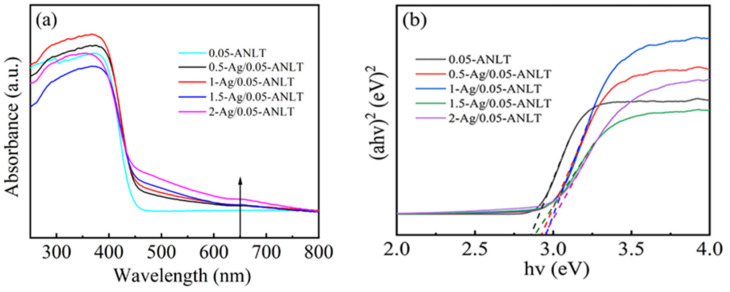
(**a**) UV-vis absorption spectra and (**b**) the estimated band gaps of 0.05-ANLT samples at different grinding times.

Generally, the noble metal nanoparticle on the semiconductors will change the light absorbance of the semiconductor through the surface plasma effect [43,44]. Figure 3a compares the DRS absorption spectra of 0.05-ANLT powder before and after grinding. Fresh 0.05-ANLT absorbs UV light up to 450 nm. However, the light absorption from 450 to 700 nm has been enhanced due to Ag nanoparticles on the surface. The increased intensity of the peak around 650 nm indicates that the Ag content rises [33]. In addition, with the increase in Ag content, SPR absorption is significantly enhanced. Generally speaking, the conduction band bottom of AgNbO_3_ is composed of O 2p and Nb 4d orbitals, but the Nb 4d orbital contributes more. Furthermore, the valence band of the AgNbO_3_ photocatalyst consists of O 2p and Ag 4d orbitals, which are motivated and generate electrons and holes under visible-light illumination. Hence, the excited state electrons mainly derive from the Nb 4d orbital of the conduction band [27]. The SPR effect from Ag NPs not only improves the visible light absorption of 0.05-ANLT but also separates photogenerated carriers, which might enhance visible light photocatalytic activity [33,45]. 

Furthermore, the band gaps (*E_g_*) of 0.05-ANLT and t-Ag/0.05-ANLT are calculated by using the following equation [46]:

(1) $${\left(Ah\upsilon \right)}^{2/n}∼h\upsilon -E_{g}$$

where *A*, *hυ*, and *E_g_* are the absorbance, the irradiation energy, and the band gap, respectively. In the context of direct and indirect semiconductors, the value of *n* is 1 for a direct semiconductor, while 4 is for an indirect semiconductor. As AgNbO_3_ belongs to a direct-gap semiconductor [27], the corresponding *n* value is 1 in present case. The *E_g_* values of the samples with 0.05-ANLT are approximately 2.87 eV (±0.03), as depicted in Figure 3b. The band gap energies of 0.05-ANLT samples after grinding for different times of 0.5, 1, 1.5, and 2 h are 2.91 eV (±0.02), 2.94 eV (±0.03), 2.89 eV (±0.04), and 2.93 eV (±0.02). These findings suggest that the band gap width of 0.05-ANLT specimens exhibits negligible variation before and after grinding due to the extremely low content of Ag. Clearly, the alteration in band gap has minimal impact on the catalytic performance of the specimen.

**Figure 4:**
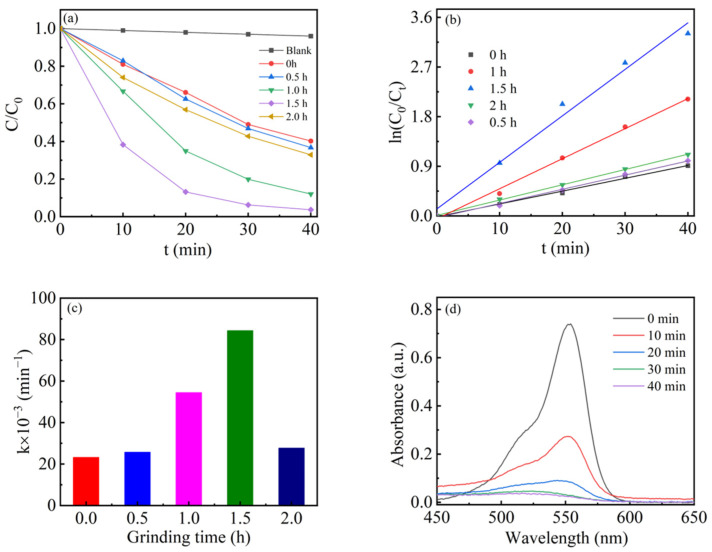
(**a**) Degradation activity of t-Ag/0.05-ANLT piezo-photocatalysts under visible light and ultrasonic excitation. (**b**) Reaction kinetics curve of t-Ag/0.05-ANLT piezo-photocatalyst. (**c**) Degradation reaction rate constants of t-Ag/0.05-ANLT piezo-photocatalyst. (**d**) UV-vis spectral absorption of RhB for 1.5-Ag/0.05-ANLT under the irradiation of light and ultrasound.

The piezo-photocatalytic degradation performance of t-Ag/0.05-ANLT for RhB under visible light and ultrasonic vibration was investigated in Figure 4. The results showed that the degradation rate was negligible without a catalyst, even when ultrasonic vibration and simulated visible light irradiation were applied. These findings suggest that the t-Ag/0.05-ANLT catalyst is crucial for the degradation of RhB. The fresh 0.05-ANLT decomposes 60% RhB within 40 min. However, compared with pure 0.05-ANLT, all t-Ag/0.05-ANLT catalysts exhibited higher piezo-photocatalytic degradation rate. The piezo-photocatalytic degradation rates of 0.05-ANLT were 60%, 64%, 89%, 97%, and 68% after mechanical milling for 0.5, 1, 1.5, and 2 h, respectively. Among them, the 1.5-Ag/0.05-ANLT catalyst exhibits the best piezo-photocatalytic performance. Obviously, the t-Ag/0.05-ANLT heterojunction effectively enhances piezoelectric photocatalytic performance.

The photocatalytic degradation of RhB follows first-order kinetics [47]. The kinetic equation can be represented as follows:

(2) $$\ln\left(C_{0}/C\right)=kt$$

where *k* denotes the apparent pseudo-first-order rate constant (min^−1^), *C* represents the concentration of the organic dye at time *t* (mol L^−1^), and *C*_0_ signifies the initial concentration of the organic dye (mol L^−1^). The degradation of the dye leads to the decoloration of the RhB solution. Figure 4b shows the degradation kinetic data, and the rate constants (*k* values) can be determined according to Equation (2). Figure 4c shows that the piezo-photocatalytic reaction rate constants of 0.05-ANLT after grinding for 0, 0.5, 1.0, 1.5, and 2.0 h are 0.02325 min^−1^, 0.02571 min^−1^, 0.54470 min^−1^, 0.08434 min^−1^ and 0.02772 min^−1^, respectively. The proper amount of Ag NPs on the surface enhances piezo-photocatalytic performance. However, further increase in Ag NPs may induce a shielding phenomenon, impeding the penetration of light radiation into 0.05-ANLT, leading to marked reduction in catalytic efficacy [48]. Figure 4d shows the absorbance spectra of RhB degraded by the 1.5-Ag/0.05-ANLT heterojunction. The gradual decrease in the absorption peak at 554 nm in Figure 4d indicates that RhB has been degraded.

**Figure 5:**
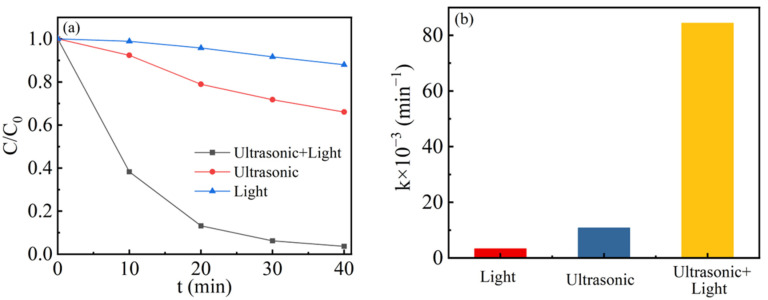
(**a**) Comparison of catalytic activities for 1.5-Ag/0.05-ANLT under different excitations. (**b**) Degradation rate constants for 1.5-Ag/0.05-ANLT under different excitations.

Degradation experiments under single light irradiation, single vibration irradiation, and the co-excitation of light and ultrasonic vibration were conducted to unveil the synergetic effect of photocatalysis and piezocatalysis. Figure 5a shows the degradation efficiency of RhB dye by 1.5-Ag/0.05-ANLT sample. After visible light irradiation for 40 min, only 13% RhB has been decomposed. Within the same time, the degradation rate increased to 35% under ultrasonic vibration. However, most RhB (97%) could be degraded under coexcitation of light and vibration. Figure 5b compares their kinetic rate constants (*k*). The degradation constants (*k*) of photocatalysis and piezocatalysis for RhB were 0.00332 min^−1^ and 0.01082 min^−1^, respectively. However, the bicatalysis rate constant sharply increased to 0.08434 min^−1^. It is suggested that a strong synergetic effect is responsible for this distinct improvement, similar with Au/AgNbO_3_ [32] and Au_x_/BaTiO_3_ [49].

**Figure 6:**
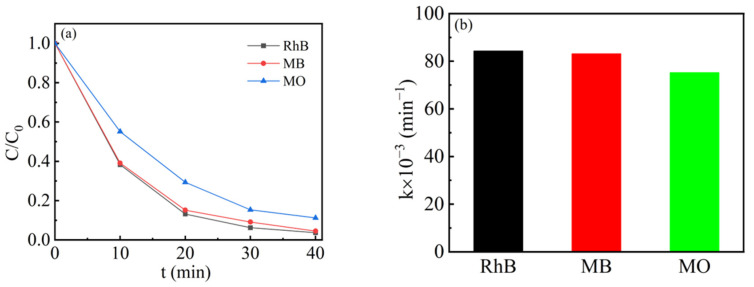
(**a**) The piezo-photocatalytic activity of 1.5-Ag/0.05-ANLT for different dyes. (**b**) Corresponding degradation rate constants.

To examine the university of the degradation capability of the 1.5-Ag/0.05-ANLT catalyst, MB and MO solutions were also degraded by 1.5-Ag/0.05-ANLT. As illustrated in Figure 6a, the degradation efficiencies of RhB, MB, and MO reached 97%, 96%, and 89%, respectively, within 40 min. Compared with MO, 1.5-Ag/0.05-ANLT has slightly higher degradation efficiency for RhB and MB dyes because their negatively charged surfaces readily adsorb the cationic dyes [50]. The degradation rate constants shown in Figure 6b for RhB and MB are 0.08434 min^−1^ and 0.08311 min^−1^, which was slightly higher than that for MO (0.07521 min^−1^). The result indicates that 1.5-Ag/0.05-ANLT can quickly decompose different dyes and work as a promising piezo-photocatalyst.

**Figure 7:**
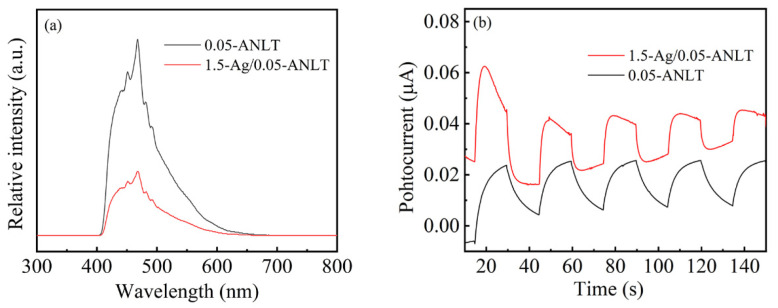

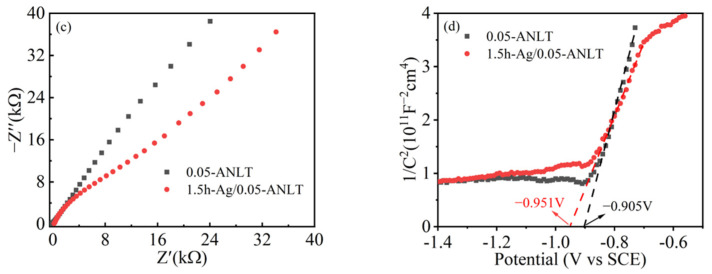
(**a**) photoluminescence spectra, (**b**) photocurrent, (**c**) EIS curve, (**d**) Mott–Schottky plots for 0.05-ANLT and 1.5-Ag/0.05-ANLT.

The PL emission occurs when electron–hole pairs recombine in semiconductor. The higher PL intensity indicates the stronger carrier recombination and lower photocatalytic activity [51]. In this study, 0.05-ANLT and 1.5-Ag/0.05-ANLT exhibit similar emission bands from 400 to 650 nm in Figure 7a. The higher intensity of the PL peak for 0.05-ANLT indicates the stronger recombination of the electron and hole. By contrast, the separation of charge carriers has been improved in 1.5-Ag/0.05-ANLT. The charge separation in photocatalysts could also be characterized by its current under light irradiation [52]. In Figure 7b, the photocurrent of the 1.5-Ag/0.05-ANLT heterojunction is higher than that of the fresh 0.05-ANLT, confirming its enhanced charge carrier separation. To further elucidate the rationale behind the enhanced piezo-photocatalytic activity, Nyquist curves have been employed to evaluate the charge migration resistance. Generally, a smaller semicircle diameter signifies lower charge transfer resistance. Figure 7c suggests that the 1.5 h-Ag/0.05-ANLT composite exhibits a smaller semicircle diameter. The Mott–Schottky in Figure 7d demonstrates that all samples belong to n-type semiconductors [53]. The flat-band potential of the 1.5 h-Ag/0.05-ANLT composite at the *x*-axis intercept is −0.951 V, more negative than that of the 0.05-ANLT composite (−0.905 V). A higher flat band potential corresponds to the stronger reducible ability of 1.5 h-Ag/0.05-ANLT composite [54].

In general, the *E_CB_* for n-type semiconductors is 0.1–0.3 eV lower than the flat-band potential value [55,56,57,58]. As Figure 7d shows, *E_CB_* values for 0.05-ANLT and 1.5 h-Ag/0.05-ANLT are −0.805 (vs. NHE) and −0.851 V (vs. NHE), respectively. The *E_g_* of 0.05-ANLT is approximately 2.87 eV (±0.03) in Figure 3b. The band gap of 0.05-ANLT samples after grinding for 1.5 h is 2.89 eV (±0.04). Based on the formula *E_CB_ = E_VB_* − *E_g_* [59], the *E_VB_* of 0.05-ANLT and 1.5 h-Ag/0.05-ANLT are 2.065 V and 2.039 V, respectively. These values are comparable with the literature [26,32]. 

**Figure 8:**
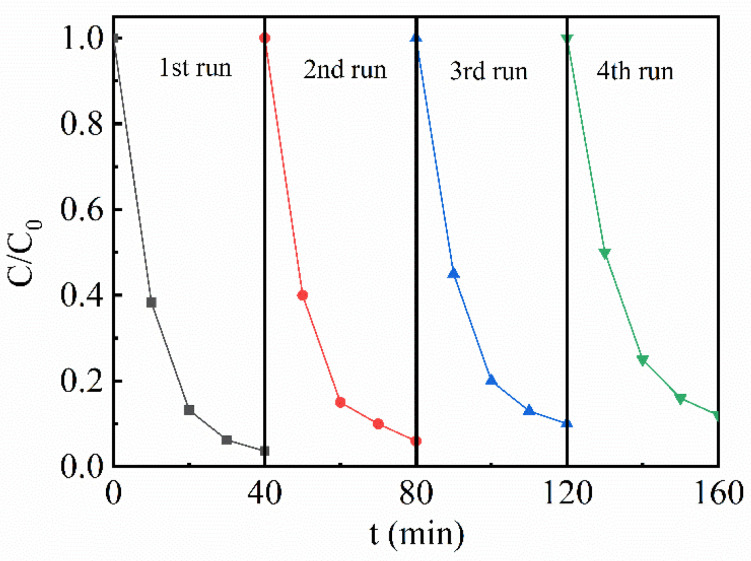
Cycling experimental of piezo-photocatalytic degradation of RhB by 1.5-Ag/0.05-ANLT.

The stability of the catalyst is very important in practical applications. The results in Figure 8 demonstrate that the t-Ag/0.05-ANLT piezo-photocatalyst still can degrade 88% RhB after four recycles. This result implies that the heterojunction shows acceptable stability. On the other hand, Yu et al. found that the XRD pattern of Ag/AgNbO_3_ by the combustion method before and after photoreduction does not change and concluded that this composite catalyst is stable in solution under light irradiation [60]. A similar process might happen in the present case, which indicates the notable stability of our catalyst.

**Figure 9:**
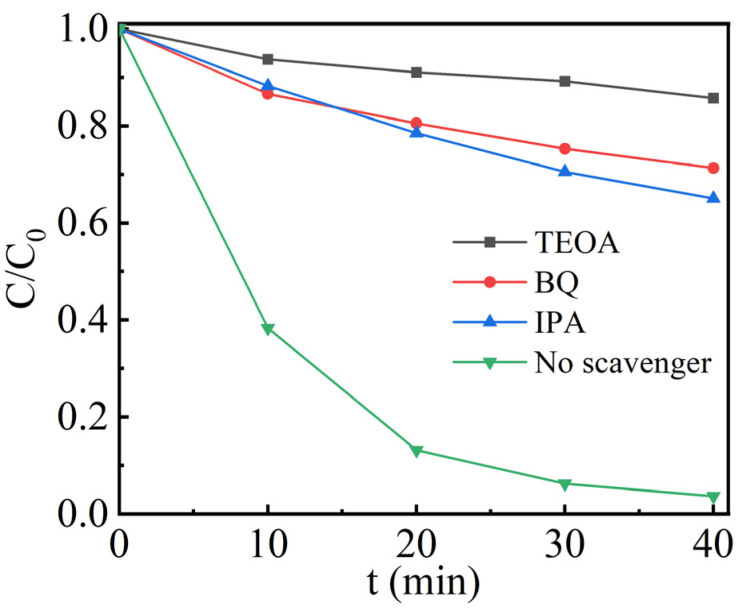
Effect of active capture agents on the piezo-photocatalytic degradation of RhB by 1.5-Ag/0.05-ANLT.

To identify the active species in the piezo-photocatalytic reaction and reveal its reaction mechanism, a series of degradation experiments of RhB have been conducted with the addition of different scavengers. Figure 9 shows that the degradation of RhB is 97% in the absence of any scavengers. However, a great reduction in the degradation rate has been observed (35%, 28% and 14%) after the addition of IPA, BQ and TEOA. Since triethanolamine (TEOA) captures *h^+^*, it indicates that *h^+^* functions as the most important active specie in the present case. However, •OH and •O_2_^−^ also play important roles during piezo-photocatalysis of RhB.

Table 1 presents some typical catalytic degradation performances of AgNbO_3_-based composite catalysts for RhB. The reaction rate constant of t-Ag/0.05-ANLT piezo-photocatalyst surpasses other AgNbO_3_-based photocatalytic materials.

**Figure 10:**
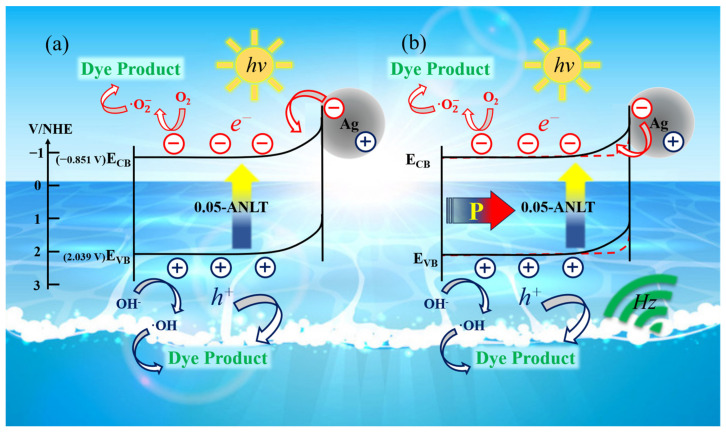
Schematic illustration of the coupled plasmonic and piezo-photocatalytic process in the t-Ag/0.05-ANLT heterostructure. (**a**) t-Ag/0.05-ANLT under visible light irradiation; (**b**) t-Ag/0.05-ANLT under the excitation of visible light and ultrasonic.

According to the active species experiment, the potential piezo-photocatalytic mechanism of t-Ag/0.05-ANLT can be proposed in Figure 10. AgNbO_3_ is an n-type semiconductor (conduction band bottom −0.805 V; valence band top 2.045 V) [26,62]. Ag has a higher work function (4.62 eV) than AgNbO_3_ (4.485 eV) [31,63]. When Ag NPs are coupled with 0.05-ANLT, the Schottky barrier forms at the metal-semiconductor interface. Upon illumination, visible light causes a collective oscillation of electrons within the Ag NPs on the surface of the t-Ag/0.05-ANLT heterojunction, which enhances visible light absorption (Figure 3) [60]. The hot electrons with high energy surpass the Schottky barrier, migrate to the conduction band of t-Ag/0.05-ANLT and leave holes in Ag Nps, suppressing the recombination of electrons and holes [32]. In addition, the piezoelectric potential under ultrasonic vibration tilts the conduction and valence bands of the 0.05-ANLT catalyst and drives electrons and holes to the opposite direction [64]. This modification, in turn, decreases the barrier height for hot electrons to transition to the semiconductor (Figure 10b). The photogenerated *e^−^* and *h^+^* in the t-Ag/0.05-ANLT solid solution are effectively separated [23]. After that, electrons on the conduction band of t-Ag/0.05-ANLT reduces O_2_ to •O_2_^−^, while *h^+^* on the top of the valance band forms highly active •OH [65]. *h^+^*, •O_2_^−^ and •OH effectively degrade the adsorbed organic dyes. Additionally, Ag NPs transfer the hot electrons to 0.05-ANLT, and thus the induced charge carriers are separated efficiently. Simultaneously, Ag NPs also act as a “fast channel”, facilitating the electron migration to the Ag/dye solution interface. All these greatly boost the generation of reactive radicals and enhance the photocatalytic performance [48]. Hence, the notable augmentation in the catalytic performance of 1.5-Ag/0.05-ANLT heterostructure should be ascribed to the contribution of the SPR effect of Ag NP and the piezoelectric potential of 0.05-ANLT.

## 4. Conclusions

Novel t-Ag NP/0.05-ANLT composite was successfully constructed using a facile mechanical milling method. Milling time has an important impact on the catalytic performance of a heterostructure. The optimized composition decomposes 97% RhB within 40 min. The SPR effect from Ag NP enhances visible light absorption of heterojunction. The decomposition activity of the optimal composition under the coexcitaion of ultrasonic and visible light has been increased by 6.8 and 24.4 times than bare piezocatalysis and photocatalysis. The injection of hot electrons from Ag NP and piezopotential from piezoelectric 0.05-ANLT greatly promotes the separation of photoinduced electron and hole, which are responsible for the distinct enhancement of catalytic activity.

## Figures and Tables

**Table 1:** Typical photocatalytic performances of AgNbO_3_-based catalyst.

Ferroelectric Materials	Pollutants	Excitation Source	Concentration of Pollutant	k × 10^−3^ (min^−1^)	Ref.
Ag/AgNbO_3_	RhB	Visible light	5.0 mg/L	44.70	[60]
Ag_2_O/AgNbO_3_	RhB	Visible light	5.0 mg/L	30.56	[27]
AgNbO_3_/AgSbO_3_	RhB	Visible light	2.5 mg/L	46.66	[61]
0.05-ANLT	RhB	Visible light + 300 W ultrsonic	5.0 mg/L	26.66	[41]
t-Ag/0.05-ANLT	RhB	Visible light + 300 W ultrsonic	5.0 mg/L	84.34	This work

## Data Availability

The data presented in this study are available upon request from the corresponding author.
